# From Zenana to courtroom: the politics of genre and gender in Sujata Massey’s historical crime fiction

**DOI:** 10.3389/fsoc.2026.1678398

**Published:** 2026-02-23

**Authors:** Nila G. T., Akaitab Mukherjee

**Affiliations:** School of Social Science and Languages, Vellore Institute of Technology, Chennai, India

**Keywords:** colonial violence, colonialism, detective fiction, hybridity, legal system, orientalism, third space

## Abstract

In the contemporary era, numerous works on the genre of detective fiction have been published by women writers. Most of the works are reflections of colonialism and its outcome and play a significant role in the formation of postcolonial consciousness. These works not only emphasise the colonial violence but also challenge to question the barrier between culture and orientalism. Sujatha Massey’s work *A Murder on Malabar Hill* (2018) anchors on colonial violence and its effect on the Indian culture and society. This article analyses Massey’s text from the perspective of colonialism. It employs Homi K. Bhabha’s theory of “third space” and “hybridity,” comprehending the protagonist’s embodiment of duality, traditional Indian culture, and the westernized legal system; a hybrid legal system in which colonial laws intersect with local customs. It thus delves into Massey’s description of mystery in the socio-political scenario of 1920s Bombay, as well as a poignant scrutiny of the struggles and upheavals taking place in colonial India.

## Introduction

Detective fiction occupies a prominent place in world literature, originating in the 1800s as a genre rooted in reason, order, and logical problem-solving (Scaggs, 2005). Early exemplars such as Edgar Allan Poe’s The Murders in the Rue Morgue (1841) and iconic characters like Sherlock Holmes (Arthur Conan Doyle) and Hercule Poirot (Agatha Christie) underscore its emphasis on evidence-based investigation and the restoration of social harmony. Todorov (1977) identifies the classic form’s two-part structure—the crime and the investigation—as embodying the Enlightenment belief in objective truth attainable through reason. Yet this clarity falters when the genre migrates beyond its European origins to postcolonial societies marked by colonial legacies, cultural hybridity, and fragmented legal systems (Mukherjee, 2003; Ghosh, 2014), where straightforward logic collides with complex local realities, compelling adaptations that integrate indigenous knowledge and perspectives. Transplanting detective fiction to such settings demands a re-examination of its foundational assumptions and conventions.

Priestman (2003) observes that the detective genre traditionally offers reassurance by having the detective restore order to a crime-disrupted society; however, in postcolonial contexts, overlapping colonial and indigenous legal systems, divergent moral frameworks, and imperial aftereffects destabilize the very concept of order itself (Mukherjee, 2019). The detective thus shifts from neutral arbiter to a figure deeply embedded in the region’s culture, society, and politics. This evolution reflects broader postcolonial literary dynamics, where, as Ashcroft et al. (1989) argue, imported genre conventions are routinely borrowed and subverted to articulate local realities, identities, and resistance. In postcolonial crime fiction, this manifests in the avoidance of clear resolutions, the questioning of the detective’s authoritative role, and the complication of simplistic notions of justice (Ten Koraar, 2005; Rzepka and Horshley, 2010). Bhabha’s (1994) concept of the third space proves particularly illuminating here: it accounts for how such genre adaptations generate ambivalent meanings and fractured power relations, thereby undermining the detective form’s foundational reliance on certainty and singular authority (Foucault, 1975).

Central to this reconfiguration is Bhabha’s (1994) concept of cultural hybridity and the third space, which critiques binary oppositions (East/West, coloniser/colonised, traditional/modern) and posits dynamic, ambivalent cultural interactions involving translation, mimicry, and resistance (Young, 1995). In postcolonial detective fiction, hybridity subverts the genre’s formulaic logic, single authoritative voice, and expectation of definitive closure (Mukherjee, 2019). Instead, it foregrounds contested histories, fragmented identities, multiple truths, and culturally situated negotiations of justice (Ten Koraar, 2005), transforming the genre into a space for articulating colonial legacies alongside native customs and hybrid subjectivities.

*A murder on Malabar Hill* (2018) revolves around Perveen Mistry, Bombay’s first female solicitor, who investigates a suspicious death in a Muslim household under purdah amid intersecting legal, cultural, and gender systems. As a Parsi woman, with an education from Oxford and working in her father’s law firm, Perveen inhabits a liminal space in colonial society: an insider within Indian communities yet an outsider to both British colonial authority and traditional gender norms. This hybrid identity equips her with the rational, evidence-based logic of Western detective traditions while granting privileged access to domestic and female centric spaces where British men are not restricted, allowing her to navigate cultural negotiations and personal insight rather than relying solely on deduction. Drawing on Grewal (1996), who examines how gender and colonial status intersect to shape women’s agency in domestic and legal spheres, Perveen’s position makes her not only an effective investigator but also a compelling exemplar of the detective genre’s postcolonial reconfiguration.

The novel merges classic detective conventions central mystery, gradual clues, and resolution with elements of historical fiction, feminist critique, and postcolonial discourse, reflecting the hybrid realities it depicts and enabling a nuanced exploration of justice and truth. Nair (1998) argues that postcolonial women writers frequently hybridize genres to challenge dominant narratives and amplify marginalized voices. Massey’s novel thus aligns with broader trends in feminist postcolonial literature that interrogate both formal conventions and societal norms.

This study argues that Sujata Massey’s *A Murder on Malabar Hill* exemplifies the postcolonial reconfiguration of detective fiction through the lens of cultural hybridity. Set in colonial Bombay, the novel deploys the protagonist’s liminal identity, the coexistence of multiple legal systems, and the intersection of gender and cultural spaces to expand rather than abandon the genre’s core conventions. By integrating diverse epistemologies, situated perspectives, and plural notions of justice, the text not only accommodates the complexities of postcolonial society but actively enlarges the detective form to reflect and interrogate the layered realities of identity, law, and power in hybrid contexts. Far from a mere adaptation, *A Murder on Malabar Hill* thus demonstrates how the genre can be transformed into a critical instrument capable of registering and challenging the multiplicities of postcolonial experience.

Colonial legal structures in India operated through legal dualism, simultaneously applying British laws and indigenous traditions, which created fragmented terrains of justice (Chatterjee, 1993; Kolsky, 2010). This fragmentation particularly complicated rights and agency for marginalized groups, including women, whose access to justice often fell between competing frameworks. Kapur (2005) underscores the value of intersectional perspectives that treat gender, culture, and law as mutually constitutive. In Sujata Massey’s *A Murder on Malabar Hill*, Perveen Mistry embodies these very tensions: as a female Parsi solicitor trained in British law, she navigates colonial legal norms while respecting indigenous religious customs and gender expectations, thereby highlighting both the constraints and possibilities within such plural legal landscapes.

Feminist postcolonial critics such as Grewal (1996), Mohanty (2003), and Nair (1998) have shown how women writers hybridize genre conventions as a form of resistance and narrative innovation, asserting female agency against patriarchal and colonial power structures; Sujata Massey’s *A Murder on Malabar Hill* extends this tradition by remaking detective fiction to articulate culturally situated, feminist modes of justice. This hybridisation further encompasses urban space, law, and gender, which intersect as dynamic forces in colonial Bombay, a city of “margins and mixture” (Kidambi, 2007) and socially produced power relations (Lefebvre, 1991; Soja, 1996). In the novel, plural legal systems, gendered norms, and hybrid spatial practices function not as mere backdrop but as active agents shaping knowledge, identity, and justice, thereby clustering spatial, legal, and gendered dimensions into a unified terrain of postcolonial critique.

Existing scholarship reveals a clear lacuna in examining how postcolonial detective fiction negotiates the intersections of genre, gender, law, and space. While critics such as Ten Koraar (2005) and Young (1995) acknowledge postcolonial resistance to dominant Western genres, their discussions of hybridity remain largely abstract, offering little concrete analysis of how it operates through narrative practices of legal investigation, gendered power relations, and spatial representation. In particular, they rarely address how the detective genre’s epistemological framework rooted in rational detection and judicial resolution is transformed in colonial or postcolonial contexts where justice is culturally contingent, thereby overlooking the material, institutional, and gendered dimensions that redefine knowledge, authority, and truth in the form.

Early Indian detective fiction adapted the Western rationalist, closure-driven model to local conditions by blending colonial police procedures with indigenous ethical frameworks, domestic spaces, community norms, caste and gender hierarchies, and plural legal systems. Kamala Sathianandhan’s *Detective Janaki* (1944) exemplifies this shift through one of the earliest Indian female investigators, who negotiates domestic expectations and public responsibility while moving between the family home and colonial institutions. The novel retains classic mystery conventions suspense, red herrings, rational explanation yet questions patriarchal and colonial authorisation of reason and speech by centring an Indian woman detective, thereby anticipating later feminist reworkings and staging conflicts between gendered norms, legal authority, and social life (Sathianandhan, 1944). Feminist scholarship offers a more incisive but underutilized lens: Grewal (1996), Mohanty (2003), and Kapur (2005) expose how colonial and postcolonial legal orders reproduce hierarchies of gender, religion, and class while constructing women as subjects governed by law rather than agents of it. Yet these analyses have seldom been extended to genre fiction such as detective stories, which inherently traverse the legal/domestic and public/private divides and thus constitute a potent though largely neglected site for interrogating gendered power within patriarchal and colonial structures. This absence marks a significant theoretical lacuna where legal, social, and narrative discourses could intersect productively (Chettur, 1940).

Similarly, while the colonial legal system in South Asia has been extensively examined by historians and legal theorists (Kolsky, 2010; Chatterjee, 1993), literary critics have paid little attention to how this system’s legal dualism the coexistence of British colonial law and local personal law shapes the representational logic of fiction. This legal plurality does not merely structure the colonial courtroom; it also informs narrative form, character construction, and the depiction of justice in literature. At the same time, spatial theorists such as Lefebvre (1991) and Soja (1996) have emphasized that space is both socially produced and politically contested, however these insights have seldom been extended to the analysis of postcolonial narrative geographies. Within colonial and postcolonial detective fiction, spatial boundaries are central to the negotiation of gender, authority, and visibility. Yet, few studies have explored how these spatial hierarchies and hybridities intersect with genre conventions to generate alternative ways of imagining justice and truth.

Sujatha Massey’s *A Murder on Malabar Hill* (2018) offers a compelling case study for examining the intersections of genre, gender, law and space. Set in 1920s Bombay, the novel revolves around Perveen Mistry, the female protagonist, who investigates a suspicious death in a confined Muslim society. Though the novel acknowledged as a commercial success, it has attracted minimal scholarly attention within postcolonial literary studies or genre criticism. Established discussions are mostly limited to general reviews, with very minimal academic attention toward the portrayal of legal pluralism, gendered spatiality, and hybrid identity. Massey’s novel, however, represents a distinctive amalgamation of historical fiction, feminist thought and classic detective conventions, providing a generative framework to explore how postcolonial genre fiction reimagines the relation between law, gender, and knowledge. Through its representation of a female solicitor, who navigates through colonial legal institutions and patriarchal domestic boundaries, the novel challenges the masculine rationality and legal authority.

This study strives to permeate this critical research gap by undertaking a detailed interdisciplinary analysis of *A Murder on Malabar Hill* (2018). Synthesizing postcolonial theory, feminist legal studies, spatial theory, and genre criticism, the research investigates the ways in which Massey’s novel reconfigures the epistemological foundations of the detective genre. It argues that the novel redefines the detective’s rational investigation into culturally distinct, ethically engaged way of investigation that reflects the multiple legal and social realities of colonial India (Mukherjee, 2016). By emphasizing the intersections of gender, law, and spatiality within the colonial cityscape of 1920s Bombay, this study expands the perception of how popular genres can be re-envisioned to express the complex epistemologies of postcolonial modernity. In doing so, it contributes to ongoing debates in postcolonial and feminist literary studies by revealing that detective fiction functions not as a derivative western form, but as a flexible, dynamic medium through which alternative visions of justice, authority, and identity are articulated.

### Conceptual framework

This research is grounded in hybridity (Bhabha, 1994) and its application through three interconnected dimensions urban hybridity, legal hybridity, and liminality to examine the interplay of space, law, and subjectivity in *A Murder on Malabar Hill*, as well as the detective genre’s transformation in a colonial–postcolonial context.

### Hybridity as a postcolonial analytical lens

Bhabha’s (1994) hybridity describes epistemic and discursive in-between spaces arising from colonial encounters, emphasizing negotiation, ambivalence, and translation over binary oppositions. It functions as a foundational principle permeating spatial, legal, and narrative structures, foregrounding the tense coexistence of modernity and indigenous traditions to generate contested sites of meaning, identity, authority, and knowledge.

### Urban hybridity: the colonial city as thirdspace

Drawing on Lefebvre’s (1991) view of space as socially produced through power relations and Soja’s (1996) Thirdspace (interplay of perceived, conceived, and lived practices), urban hybridity analyses Bombay’s spatial ambiguities—neither purely traditional nor fully modern. Kidambi (2007) characterizes the city as one of “margins and mixture,” where imperial, capitalist, and native elements intersect. In the novel, this hybrid urban field shapes narrative form, gendered access, and justice negotiations, transforming the detective’s role into spatial decoding influenced by gender, class, religion, and colonial authority.

### Legal hybridity: colonial law and indigenous personal codes

Legal hybridity refers to the coexistence and interplay of multiple frameworks within one jurisdiction. Following Kolsky (2010), colonial India operated via imperial statutory law alongside native personal laws (marriage, inheritance, family). This pluralism served colonial rule rather than diversity, creating uncertainty—especially for women constrained by customs like purdah. Feminist legal theory (Kapur, 2005) underscores how legal outcomes are inseparable from gendered power; the novel illustrates this through Perveen’s navigation of dual systems as negotiation rather than fixed authority.

### Liminality and the interstitial subject

Liminality denotes existence “between and among” social classifications (Turner 1969), where Bhabha’s (1994) postcolonial subject operates amid conflicting meanings. Perveen Mistry embodies this: a Parsi woman, Western-educated lawyer, and colonial subject with conditional access to institutions and domestic spaces. Her liminal position enables agency through contextual insight rather than universal reason.

### Integrative framework: hybridity, justice, and genre

Together, these dimensions form a cohesive lens: urban hybridity sets the spatial context, legal hybridity the normative terrain of justice, and liminality the subjective standpoint. This reframes detective fiction as negotiation rather than singular truth-seeking. In *A Murder on Malabar Hill*, justice emerges via translation, ethical sensitivity, and contextual reasoning, making hybridity both object and method for interrogating colonial modernity, gendered power, and postcolonial epistemology.

## Method

This study uses a qualitative research approach, specifically interpretive literary analysis, to examine how Sujata Massey’s *A Murder on Malabar Hill* changes detective fiction conventions using postcolonial and feminist perspectives. Qualitative research is appropriate here because it values in-depth analysis over broad surveys, emphasizing the interpretation of meaning, context, and representation crucial to literary and cultural studies. The novel is considered not only a work of fiction but also a cultural artifact that reflects on and critiques the social, legal, and spatial aspects of colonial India.

### Methodological approach

The study draws on several interdisciplinary critical theories such as postcolonialism, feminist legal studies, spatial theory, and genre analysis. These theoretical lenses do not function separately; they are blended together for a more complete interpretation of the novel. The analysis of hybridity and identity, and power dynamics in colonial Bombay, draws on postcolonial theory, especially the writings of Homi Bhabha. The gendered relations of law, power, and justice are explored through feminist legal theory as advanced by Ratna Kapur and Chandra Talpade Mohanty. The physical and symbolic spaces, for example, the zenana, the courtroom, and the colonial city, are regions through which one may access knowledge, movement, and agency, and are spatially dominated by Lefebvre and Soja. Genre theory, particularly from critics like Tzvetan Todorov and John Scaggs, guides the analysis of how Massey adapts and subverts detective fiction conventions to accommodate postcolonial and feminist themes.

### Researcher’s reflexivity

This interpretive analysis is shaped by my theoretical adherences to postcolonial and feminist frameworks, which foreground hybridity, intersectional gender dynamics, and situated justice in colonial contexts. As a researcher engaging with transnational literary studies, we prioritize these lenses to emphasise Perveen Mistry’s subversive power. Although, We acknowledge that possible approaches such as formalist genre analysis, Marxist readings of class in colonial Bombay, or trauma theory might establish different textual elements like, economic exploitation or psychological fragmentation and produce contrasting ideas. This reflexivity accentuates the constructed, partial nature of all interpretive claims and enhances the study’s epistemological transparency.

### Materials

This study uses Sujata Massey’s novel, *A Murder on Malabar Hill* (2018), as its main source. As the first book in Massey’s Perveen Mistry series, this story, which takes place in 1920s Bombay, provides a lot to work with when looking at changes in genre, how women take action, the mix of different legal systems, and how spaces are dealt with. The book was picked because it tells a complicated story, is based on history, and because it knowingly changes the usual detective story using postcolonial and feminist ideas. Its setting, characters, and legal problems are all good starting points for seeing how books can interact with and judge society and politics.

To back up this reading, the study also uses a number of academic sources. These include writings on postcolonial studies (like Homi Bhabha’s The Location of Culture), feminist legal theory (including work by Ratna Kapur and Chandra Talpade Mohanty), spatial theory (especially Henri Lefebvre’s The Production of Space and Edward Soja’s Third-space), and genre criticism (such as Tzvetan Todorov’s The Typology of Detective Fiction and Martin Priestman’s work on crime stories). These books give us the ideas needed to understand the book’s form and what it talks about within its historical and theoretical background.

Other sources include historical books and legal writings that give background on the different legal systems in colonial India. They show how British law existed next to local religious laws. These sources help connect the book analysis to the real-world conditions of the time the book describes. For example, studies of the Bombay High Court, the Parsi legal world, and how colonial governments worked are used to understand the legal and cultural situations that Perveen Mistry deals with. Together, these sources create a base for a detailed study of *A Murder on Malabar Hill*. This lets the study go past just reading the book and move into a mix of different fields to look at how books can be a place for cultural critique and changes in genre.

### Procedure

This study adopts a qualitative, interpretive approach using close reading and thematic analysis to explore how *A Murder on Malabar Hill* changes detective fiction conventions through postcolonial and feminist perspectives. The process includes several connected steps, each designed to examine the novel’s textual, structural, and contextual parts in a structured way. First, the novel is read to grasp its storyline, main characters, and main ideas. In this step, notes are taken on how the novel shows legal systems, settings, cultural habits, and gender roles. This reading also points out parts of the book that seem to change or redo typical detective fiction elements, like the detective’s job, the role of law, and how the crime is solved.

After the first reading, the second step includes a more detailed, theory-aware, close reading of chosen sections. This section relies on critical ideas from the methodology, like postcolonial theory, feminist legal theory, and spatial theory. It looks at Perveen Mistry as a hybrid person, legal issues across different systems, gender and space in the courtroom and zenana, and how colonial Bombay is built through senses and symbols, all through the lens of these theories. The third phase combines literary analysis with historical and cultural background research. Materials about colonial legal practices in India, gender roles in Bombay in the early 1900s, and the architecture and social layout of colonial urban areas are reviewed to add depth to the interpretation. This background makes sure the analysis is practical and stays connected to the real-world situations shown in the text, instead of just being theoretical.

Throughout this work, analytical notes are grouped by theme, with findings sorted under headings like “Legal Hybridity,” “Gender and Law,” “Space and Power,” and “Genre Subversion.” This allows for a detailed and unified final interpretation. The themes are looked at for how they come together, especially where spatial, legal, and gendered structures intersect to shape the story and the search for justice. In the end, the research comes together in a synthesis phase. Here, the information from both textual and background analysis is interpreted together to support the main point: that *A Murder on Malabar Hill* changes detective fiction to include and critique the complexities of postcolonial identity, legal diversity, and gendered space. This synthesis is used to construct the final chapters or sections of the study, drawing connections between literary form and social critique.

## Result

This study has explored how *A Murder on Malabar Hill* reconfigures the detective fiction genre through a complex interplay of spatial, legal, and cultural hybridity. The findings of this research demonstrate that Sujata Massey’s novel not only subverts the conventions of classic Western detective fiction but also reconstructs the genre as a vehicle for articulating postcolonial feminist concerns, particularly in the context of colonial Bombay’s plural legal and socio-spatial systems.

One of the most significant findings is that space in the novel functions as an epistemological and political force, rather than as a passive backdrop. The spatial arrangement of 1920s Bombay, which included British structures, local residences, and gender-specific areas like the zenana, shapes the framework and execution of this study. Crime scenes in the book are culturally embedded, requiring both analytical reasoning and contextual understanding. This questions the usual idea in detective stories that a crime scene is a simple, open space for investigation.

Also, the book puts legal mixing in the front as both a story device and a theme. By showing Perveen Mistry dealing with British laws and Islamic rules, the book shows the disagreements and disputes that come with colonial legal systems. The narrative dramatises the disjunction between codified colonial justice and lived cultural practices, revealing the ways in which colonial governance used legal dualism to maintain control while appearing to preserve indigenous customs. Perveen’s legal work demonstrates that justice in a hybrid society cannot be achieved through universal principles alone; it must be locally situated, ethically negotiated, and culturally fluent.

The study also finds that Perveen Mistry’s hybrid identity Parsi, Western-educated, female, and legally trained, is both a tool of empowerment and a site of vulnerability. Her positionality allows her to move across boundaries that male or colonial actors cannot access, such as the private spaces of Muslim women in purdah. At the same time, she remains an outsider in many contexts, navigating suspicion and marginalization from both colonial and indigenous patriarchal structures. This liminality enables her to act as a cultural intermediary, translating between competing systems of meaning and facilitating justice in ways that transcend institutional limitations.

The study of the genre shows that *A Murder on Malabar Hill* changes the detective figure from a rational authority to one who dialogs and empathizes. Instead of detectives like Holmes or Poirot, who use deduction and detachment, Perveen uses culturally aware strategies, listens closely, and connects relationally. Her way of doing things fits with feminist ideas that value specific knowledge and ethical responsibility over general ideas and certainty.

The research also indicates that Massey’s story offers a wider political and literary analysis. It questions the impact of colonialism using the plot, characters, and the way the genre is rethought. By placing the detective story in a detailed postcolonial setting, Massey shows the limits of Western genre rules and the ability of stories to act as a place for resistance, discussion, and fairness.

The data suggests *A Murder on Malabar Hill* goes beyond typical historical crime fiction, engaging with postcolonial ideas, feminist legal perspectives, and genre evolution. It is an example of how detective stories can be changed to deal with complicated issues of identity, law, and space in postcolonial societies.

### Analysis

#### Spatial hybridity: producing the colonial city

Massey constructs 1920s Bombay as a palimpsest of competing histories, where colonial laws and indigenous life alters one another. This hybridity is not simply atmospheric, it is entrenched in the narrative through recurring scenes where Perveen must understand the city’s spatial contradictions. In one of the earliest descriptions, “Perveen glanced out the window and down to the street. Fort’s twenty square miles were once the East India Company’s original fortified settlement. Now the district…British and Hindu and Muslim law offices… Indian- born Zoroastrians. Although Parsis accounted… they constituted one- third of its lawyers” (Massey, 2018, p. 10). This passage layers four distinct spatial identities, i.e., British, colonial-law, Parsi, and Irani within a particular locality. In Lefebvre’s (1991) terms, space here is produced through overlapping systems of power, economy, and culture; the city is not a static container but a living product of these frictions.

“Perveen glanced out the window and down to the street. Fort’s twenty square miles were once the East India Company’s original fortified settlement. Now the district was known for the High Court and the many law offices around it. Nestled alongside the British and Hindu and Muslim law offices were a significant number owned by members of her own religious community, the Indian-born Zoroastrians. Although Parsis accounted for just 6 percent of Bombay’s total inhabitants, they constituted one-third of its lawyers.

Iranians the Zoroastrian immigrants who had come from the nineteenth century onward prided themselves on running superlative bakeries and cafés serving cuisine influenced by their ancient homeland of Persia. Such was Yazdani’s, the bakery-café across the street. The shop drew more than 200 customers every day. This morning, the customers going in and out were working their way around a solitary obstacle” (Massey, 2018, p. 11).

The syntax of the passage itself reflects hybridity: colonial institutions (British offices, law chambers) are immediately followed by local spaces of leisure and community (Parsi cafés, Irani bakeries). This narrative sequence dissolves hard boundaries between coloniser and colonised, producing what Kidambi (2007, p. 67) calls a “city of margins and mixture”. Significantly, Massey does not describe these as separate zones rather as abreast presence co existing yet never fully mingle, whose tension defines the everyday movement of the city.

Massey continues this spatial layering through sensory detail. Perveen sits in Sir David Hobson- Jones’s car as it moves through Bombay “the car pulled up at a very tall gate that overshadowed by a giant vanilla- colored bungalow. Four guards rushed forward, a pair saluting the car while other opened the gate” (Massey, 2018, p. 69). These juxtapositions positions Bombay as a sensory third space, not merely a physical terrain but a space in which cultural and political meanings are constantly negotiated (Soja, 1996). The stiff posture of the British officer contrasts with the fluid rhythms of the servants or the gatekeepers. This contrast performs hybridity rather than merely describing it.

Such spatial hybridity shapes Perveen’s professionalism. When she travels through these mixed social circle, her task is not only to gather leads but to interpret the sociocultural logics embedded in those spaces. For example as she studies the Muslim nawab’s bungalow from Alice’s pink, sea-facing bedroom, Perveen notices “greenish mildew had bloomed in places… the door creaked open on its heavy, rusted hinges. A boy stood… wearing a shabby vest and pantaloons” (Massey, 2018, p. 82). This visual metaphorically articulates Bombay’s epistemic hybridity.

Perveen’s journey through this scene enacts this hybrid epistemology. She listens how Lady Hobson- Jones’s remark that “Muslims keep to themselves” and replies that “it depends on the family” (Massey, 2018, p. 73). She immediately personifies the decaying bungalow on the sea view road to the Farid widows. She describes the Malabar hill as a stratified landscape, how one house shelters the elite British power, i.e., the house of Alice, another houses a wealthy manager while the “miniature ivory palace” below secludes the widows of Farid. The coexistence of these residential forms reveals, in Soja’s term, a *trialectic* of space: physical (the architecture), social (the servants, neighbors and women in purdah), and mental (colonial dream of real India versus Indigenous world). Perveen’s ability to interpret both speaks to her hybrid identity.

This interpretive skill becomes central to the novel’s critique of Western detective rationality. In classic detective fiction, the crime scene is presumed to be a neutral site awaiting objective investigation. Massey explicitly subverts this neutrality. When Perveen is lured out of her Bruce street office by an anonymous call for help and discovers that “someone dropped nails and broken glass on both ends of the street” (Massey, 2018, p. 378). The path itself constructed a trap rather than a transparent conduit to the facts. The abduction, in which she is covered in the “scratchy sack” and “the crowding gave the impression she was in a storeroom: perhaps one of the many godowns built in rows near the harbor” actively creates what can be seen, said or to be proven in law. The space itself becomes a clue: its structure signals the presence of an entirely different cultural logic governing visibility, gender, and access.

Thus, spatial hybridity becomes both narrative device and theoretical argument. The hybrid locality demands hybrid investigative methods. Perveen cannot only rely on British legal training, she must have a knowledge of architectural cues, sensory landscapes, and cultural geographies. Her movement across these varied spaces demonstrates Massey’s claim that truth in colonial Bombay is never singular or objective. It must be pieced together from within the “in-between” spaces of everyday life.

### Malabar Hill and the architecture of the third space

Malabar Hill Bombay’s affluent enclave functions not merely as a geographical location but as an architectural embodiment of cultural hybridity. When Perveen first sees the Farid residence from Alice Hobson-Jones’s bedroom, she notes a “low cream-colored house” built in an “Indo-Saracenic bungalow with gardens on its sides and central garden courtyard with a long, rectangular pool” (Massey, 2018, p. 73), visually distinct from the neo-Georgian bungalows flanking it on the hill. This contrast, in which a rotting “miniature ivory palace” sits literally in the trails of British and Parsi bungalows, directs what Bhabha (1994) identifies as the Third Space, the site where two cultural systems meet, neither erasing nor fully assimilating the other.

The architecture is itself an argument. The British mansions around the Farid property, with their “mahogany stairs that curved gently as they rose… tall bay windows… and sea views”, exhibits the prestigious society and modernity associated with the coloniser, while the Farid bungalow appears more “Indian,” its peeling “stucco” and “the glass spiked” wall marking it as both adjacent with and apart from this county of imperial power. Yet once Perveen begins working on the Farid estate, she learns that the widows live as “purdahnashins”, women who “stay behind the veil,” occupying a strictly secluded buildings called zenana where men are restricted. The zenana therefore performs a dual identity: outwardly plain within Malabar Hill’s colonial landscape, inwardly organized according to Islamic gender norms and seclusion. The Farid household is thus a physical demonstration of colonised elite’s aspirations and anxieties, a hybrid environment where class mobility, religious orthodoxy, and the desire for respectability co-exist.

### Architecture as power

For the detective narrative, this architecture does not serve merely as backdrop; it systematically shapes what can be known, by whom, and under what conditions. When Perveen discusses the widows’s mahr with her father, he explains that as “purdahnashins” they “don’t speak with men,” (Massey, 2018, p. 22) so he has never met them despite years of representing Mr. Farid, which means only a woman lawyer can enter their side of the house and obtain their testimony. The walls, jali screens, and private women’s quarters thus function as technologies of gendered power, regulating visibility and controlling which voices can reach the colonial legal system. This aligns with Lefebvre’s (1991) theory that built environments encode social hierarchies. The Farid bungalow enforces seclusion not through ideology alone but through material arrangements gated boundaries, divided sections, and interior spaces that men like Jamshedji and the police cannot legitimately cross.

Perveen’s ability to access this space is therefore not simply procedural; it is a culturally mediated forensic act. Her status as both solicitor and respectable woman allows her to cross from public, colonial exteriors into the zenana, where she can speak directly with Razia-begum and the other widows. A male detective would be barred at the gate, forced to rely on intermediaries like Mukri, whose forged signatures and secret dealings the novel exposes as deeply unreliable. The space, in other words, denies their access and thereby redistributes epistemic authority toward Perveen and the women whose stories she hears.

### The Zenana as an epistemological space

When Perveen converses with the widows “behind the curtain,” she becomes acutely aware that the architecture itself is communicating meaning. “She could not distinguish anything except the fact there were three voices” (Massey, 2018, p. 240). She cannot see the women clearly, but she can distinguish their different voices, hesitations, and levels of literacy as they discuss the wakf and mahr, so that tone, silence, and the very act of signing or refusing to sign become key evidentiary clues. Here, Massey shifts the method of detection away from the visual evidence typical of Western detective fiction toward sound, silence, gesture, and spatial cues. Knowledge emerges not from rational deduction alone but through reading *the space*.

This aligns precisely with Soja’s (1996) concept of Third spac*e* as an epistemological zone a site where understanding is shaped by cultural translation. The zenana is neither fully private nor fully accessible; it is a permeable boundary where truth is relayed indirectly, filtered through spatial norms. The widows cannot appear before Perveen, but they can modulate tone, movement, and silence. Their testimony is spatially mediated.

Thus, the architecture does not simply contain the mystery it produces it. Every clue is entangled with spatial codes: who is permitted to enter, who must remain hidden, who speaks but cannot be seen. Perveen’s investigation must therefore move beyond Western forensic norms, requiring her to interpret the non-visual, the partially concealed, the culturally encrypted.

### Between purdah and the courtroom: identity as hybrid practice

Perveen Mistry travels through Bombay’s hybrid spaces because she herself represents as a product of hybridity. “*Bombay Samachar* touting her as Bombay’s first women solicitor” (Massey, 2018, p. 12). She is introduced as identities that position her between communities and institutions. Her Oxford education gives her authority in the colonial judicial system, yet her gender and ethnicity restrict her within patriarchal norms; she is welcomed and mistrusted simultaneously.

This liminality corresponds to Bhabha’s (1994) figure of the *interstitial subject*, one who inhabits the “in-between,” neither fully colonial nor fully indigenous. When Perveen states that she can speak with the widows only “behind the screen” (Massey, 2018, p. 87), her position reveals both access and exclusion. She is allowed entry because she is a woman; she is trusted because she respects Islamic customs; she is effective because she understands British legal codes. Her identity bridges across cultural, legal, and gendered divides.

Mohanty (2003) argues that postcolonial feminist agency emerges not from escaping oppression but from negotiating multiple oppressions simultaneously. Perveen’s agency is precisely this: she navigates patriarchy at home, communal expectations within the Parsi community, and colonial hierarchies in court, even as she advocates for dockworkers like Jayanth and purdahnashin widows against powerful employers and guardians. For example, when Perveen meets the Farid widows in the zenana, she attends closely to the nuances of their speech, differing levels of literacy, and emotional states as they discuss the wakf and their mahr, noticing “Razia’s tone was reproving. Perveen sensed the widows were anxious about her presence” (Massey, 2018, p. 102). Later, in private conversations with each woman, she reads hesitation, sudden outbursts, and shifts in confidence as evidence of coercion and fear, understanding that seclusion, mourning, and economic dependence shape what they can safely say. This method exemplifies Haraway’s (1988) idea of situated knowledge, all knowledge is partial, contextual, and shaped by the specific time, place, culture and embodiment of knower. Perveen’s understanding arises from attentive listening to embodied, context-specific experiences inside purdah, not from an abstract, universal vantage point.

### Legal hybridity: navigating dual systems

Bombay’s hybridity in *A Murder on Malabar Hill* extends decisively into the judiciary sphere, where law itself emerges as a contested and unevenly accessible terrain. The Bombay High Court, where Perveen practices as a solicitor, operates as a hybrid institution that simultaneously enforces British civil law and defers to Hindu, Islamic, and Parsi personal laws in matters of marriage, inheritance, and succession. This arrangement exemplifies what Kolsky (2010) defines as legal dualism: a colonial strategy that claims respect for indigenous traditions while ultimately preserving imperial authority through selective interpretation and enforcement.

“Perveen read the letter from the appointed estate trustee, Faisal Mukri. Mr. Mukri wanted her to make a change that would disrupt the estate settlement on which she’d been working” (Massey, 2018, p. 12).

Here Massey repeatedly underscores the instability of this system through narrative detail rather than abstract exposition. In the Farid inheritance case, “Mr. Mukri had written that all the widows wanted to give up their assets as donations to the family’s wakf, a charitable trust that provide funds each year to the needy while paying a dividend to specified relatives” (Massey, 2018, p. 12). The ambiguity is not incidental; it enables male guardians to exploit the gap between Islamic succession law and British probate procedure. As Ratna Kapur argues, feminist engagements with law in postcolonial contexts must attend to the ways in which legal regimes, including religious personal laws, are mobilized to reproduce gender and cultural essentialisms and to discipline women’s claims rather than expand their agency (Kapur, 2005).

Perveen’s role therefore exceeds that of a conventional legal practitioner. Perveen’s role therefore exceeds that of a conventional legal practitioner. She becomes what Kapur would call a postcolonial feminist legal actor, one who negotiates between competing vocabularies of culture, religion, and rights rather than assuming that more law automatically equals more freedom (Kapur, 2005). Her position as Parsi, Western-educated, and female allows her to translate between British probate procedure and the gendered constraints of Muslim personal law in a way that neither colonial judges nor male guardians can This mediation is most powerfully dramatized in her conversations with the Farid widows conducted “from behind the screen” (Massey, 2018, p. 87). One widow recounts, in “a voice barely above a whisper,” that they were made to sign documents they “were not permitted to read” and that “hands we trusted placed the pen where we were told” (Massey, 2018, p. 98). The phrasing foregrounds enforced passivity and coerced consent, revealing how legal acts become hollow rituals when gendered restrictions deny women meaningful participation.

Here, Massey exposes how law is spatially and bodily regulated. The widows’ inability to appear in court, speak openly, or even view the documents governing their property rights demonstrates how personal law, purdah, and colonial bureaucracy converge to produce systematic exclusion. Justice is theoretically available but practically unreachable. This narrative dynamic aligns closely with Kapur’s (2005) feminist legal critique that law cannot be analyzed apart from its entanglement with gender, culture, and power. Massey’s fiction gives narrative form to this argument by showing that the law’s promise of protection collapses when legal subjects are rendered invisible by custom and misunderstood by colonial institutions.

Perveen’s intervention resists both colonial universalism and patriarchal manipulation. Rather than privileging one legal system over another, she works within the fractures between them. She listens to the widows’ testimonies, reconstructs the circumstances of the will’s signing, and anticipates how British courts will misinterpret or ignore religious and domestic constraints. Her legal reasoning is therefore dialogic and contextual, not deductive. It emerges from what she learns “in the half-light of the women’s quarters,” not solely from casebooks or statutes (Haraway, 1988).

Through this portrayal, Massey critiques the foundational assumption of classic detective fiction: that law provides a stable endpoint for truth and justice. In *A Murder on Malabar Hill*, legality is provisional, negotiated, and ethically fraught. Resolution depends not on uncovering a single truth but on navigating competing normative systems. The detective’s task becomes inseparable from legal interpretation, cultural literacy, and feminist advocacy.

Ultimately, legal hybridity in the novel is not presented as a harmonious coexistence of systems but as a lived tension that demands interpretive labor. By positioning Perveen as a hybrid legal actor Parsi, female, Western-trained, culturally fluent Massey demonstrates how justice in a colonial society can only emerge from within the cracks of law itself. Fiction thus becomes a critical site for exposing the limits of colonial legality and imagining a more situated, relational form of justice.

### Narrative hybridity and the challenge to the detective genre

Through its sustained engagement with hybrid spaces, hybrid identities, and hybrid legal systems, *A Murder on Malabar Hill* does not merely adapt the detective genre to an Indian setting; it fundamentally restructures the genre’s epistemological assumptions. Classical detective fiction is premised on a linear progression toward a singular, rationally apprehensible truth, culminating in a definitive act of revelation. Massey deliberately resists this model. The novel’s resolution emerges instead through a series of negotiations between colonial and indigenous law, public and private space, speech and silence revealing justice as an ethical process rather than a purely deductive outcome.

This resistance to closure is visible in the novel’s narrative pacing and structure. Crucial information is disclosed not in moments of dramatic confrontation but in fragmented exchanges, whispered testimonies, and delayed recognitions. When Perveen concedes that “there were answers the law would never hear,” the novel refuses the detective genre’s fantasy of exhaustive revelation. Haraway’s critique of the “view from nowhere” is instructive here: knowledge, including legal knowledge, is always partial and located, never total (Haraway, 1988). Massey’s refusal of full closure thus aligns detection with feminist epistemology rather than with positivist certainty.

Bombay itself functions as both metaphor and methodological framework for this reconfigured detection. The city’s spatial heterogeneity its overlapping colonial boulevards, crowded bazaars, domestic interiors, and judicial chambers mirrors the narrative’s movement across incompatible yet coexisting epistemic systems. Perveen’s investigative path follows this spatial logic. Her journey from the Bombay High Court to the secluded zenana is not merely geographical but epistemological, marking a shift from codified legality to embodied, experiential knowledge.

This parallel is made explicit in Perveen’s self-reflexive observation that “Bombay was a city that never slept, always changing, yet always the same like me” (Massey, 2018, p. 310). The simile collapses the distinction between subject and space, rendering identity itself as spatially produced. Perveen, like the city, is composed of temporal layers—colonial education, indigenous belonging, feminist consciousness—held together through continuous negotiation. This moment enacts Lefebvre’s (1991) assertion that social space is not a passive container but a material expression of historical contradiction. Bombay’s contradictions modernity and tradition, imperial authority and indigenous resilience are inscribed onto Perveen’s body and consciousness.

Massey’s use of the city thus exceeds descriptive realism and operates as a theoretical intervention. Bombay becomes what Soja (1996) would term a *trialectical space*, where physical form, social practice, and imaginative meaning intersect. The city does not simply host the detective narrative; it generates the conditions under which detection must occur. The impossibility of a singular resolution mirrors the city’s refusal to be reduced to one identity or logic. Just as Bombay cannot be fully mapped, the case cannot be fully closed.

In reimagining detection as a practice of translation rather than revelation, Massey aligns the genre with postcolonial epistemologies that privilege multiplicity, relationality, and ethical attentiveness. Perveen’s final actions emphasise care over triumph, responsibility over mastery. Her justice is provisional and context-sensitive, attentive to the lives affected by legal decisions rather than to the satisfaction of narrative closure. This approach resonates with feminist critiques of objectivity, particularly Haraway’s (1988) concept of situated knowledge, which rejects the “view from nowhere” in favor of accountable, partial perspectives.

By embedding investigation within Bombay’s layered spaces and fractured legal systems, Massey demonstrates that truth in a colonial context is not discovered but negotiated. The novel thus reframes detection as an ethical and interpretive practice one that recognizes the limits of law, the weight of history, and the necessity of listening across difference.

## Discussion

The findings of this study reveal that Sujata Massey’s *A Murder on Malabar Hill* reimagines detective fiction through a richly hybridized postcolonial and feminist lens, disrupting the genre’s traditional reliance on rationalism, linear justice, and authoritative closure. This discussion aims to critically interpret these findings in light of the theoretical frameworks underpinning the study: postcolonial theory, feminist legal studies, spatial theory, and genre criticism. It examines how Massey’s novel not only reflects the socio-legal and cultural complexities of colonial Bombay but also enacts a literary intervention that expands the boundaries of genre and knowledge production.

A central argument arising from the analysis is that Massey’s depiction of spatial hybridity functions as both a literal and metaphorical representation of postcolonial negotiation. Drawing on Lefebvre’s (1991) concept of space as socially produced and Bhabha’s (1994) notion of the “Third Space,” the novel portrays colonial Bombay not as a static backdrop but as an active participant in the construction of meaning, authority, and identity. The juxtaposition of British colonial institutions and traditional Indian domestic spaces, such as the courtroom and the zenana, foregrounds the city’s layered, contested nature. Perveen Mistry’s ability to navigate these spaces is emblematic of her hybrid identity, which grants her unique epistemological access to truths that remain obscured to more institutionally privileged but culturally disconnected actors.

This spatial hybridity is intricately connected to legal pluralism, another key theme the novel explores. As Kolsky (2010) argues, colonial legal systems operated on the logic of dualism, simultaneously imposing imperial law and preserving indigenous legal traditions as a means of control. Massey critiques this framework by illustrating how legal structures, when divorced from cultural understanding, fail to deliver justice, particularly for women. Perveen’s investigation of the Farid case demonstrates that legal reasoning in colonial India could not be purely technical or procedural; it had to be interpretive, ethical, and culturally embedded. In this way, the novel aligns with feminist legal theorists such as Kapur (2005), who emphasise the need for law to engage with the intersectionality of gender, culture, and social identity.

Moreover, Perveen’s hybrid identity as a Parsi woman trained in English law but deeply rooted in Indian culture challenges both colonial patriarchy and indigenous orthodoxy. Her ability to move between Western legal spaces and traditional domestic settings allows her to act as a cultural translator. This mediating role resonates with Bhabha’s concept of the “interstitial subject,” who occupies the in-between spaces of colonial discourse, challenging fixed categories and binary oppositions. However, Massey does not romanticize hybridity; she also highlights its burdens. Perveen’s marginalization in both colonial institutions and conservative Indian society illustrates that hybridity is often fraught with tension and vulnerability. Massey’s diasporic standpoint further enriches this portrayal, allowing her to depict hybridity not as abstract theory but as lived, ambivalent experience informed by her own bridging of Indian heritage, Western education, and global mobility.

The reconfiguration of the detective figure is another significant point of discussion. As analyzed earlier, Perveen Mistry embodies a situated, rational epistemology that subverts the genre’s rationalist authority as established previously. Her method is grounded in empathy, active listening, and cross-cultural negotiation. This aligns with feminist critiques of traditional epistemology, such as those articulated by Haraway (1988), which argue for the value of “situated knowledges” over universalist claims to truth. Perveen’s investigative process reflects a feminist ethics of care, particularly in how she handles sensitive issues involving women in purdah and in how she balances legal principles with cultural and emotional realities.

Genre-wise, *A Murder on Malabar Hill* serves as a meta-commentary on detective fiction itself. By refusing to treat crime scenes as neutral and by disrupting the linearity of investigation and resolution, Massey challenges the foundational assumptions of the genre. The novel does not offer a clean closure that restores social order in the manner of classic detective stories. Instead, it presents justice as contextual, negotiated, and provisional, reflecting the complexity of life in a plural, postcolonial society. This genre subversion aligns with scholars like Ten Koraar (2005), who argue that postcolonial adaptations of detective fiction tend to resist finality and expose the instability of truth in colonised settings.

Furthermore, the novel’s critique of both colonial and indigenous patriarchal systems positions it within the broader tradition of postcolonial feminist writing. As Mohanty (2003) notes, feminist analysis in postcolonial contexts must be attentive to the interplay between gender, culture, and imperial power. Massey’s representation of Perveen as both empowered and constrained illustrates the ambivalence of agency within overlapping systems of domination. The fact that Perveen’s authority is derived not from power but from her ability to negotiate boundaries challenges masculinist portrayals of detective work and highlights alternative forms of agency.

Beyond its literary reconfiguration of detective fiction, this analysis contributes to sociological debates on race, gender, coloniality, and legal institutions. The novel dramatizes how colonial legal pluralism (Kolsky, 2010) intersected with gendered and racialized hierarchies, e.g., Parsi women’s liminal status, purdah’s spatial exclusions, and women’s limited agency under personal laws. By foregrounding situated justice through Perveen’s negotiations, Massey’s text illuminates mechanisms of institutional hybridity and resistance, complementing empirical sociological studies of postcolonial law, intersectional inequality, and racialized legal subjects in colonial India. This bridges literary representation with sociological understandings of how fiction mediates and critiques power structures in plural societies.

The discussion confirms that *A Murder on Malabar Hill* is a multi-layered work that examines historical, legal, spatial, and gendered injustices through the detective fiction genre. The novel transforms genre conventions to make room for hybrid subjectivities, plural legal systems, and culturally situated practices of justice. Massey’s narrative does not merely insert a female protagonist into a colonial setting; it rethinks what it means to investigate, to know, and to judge within a world shaped by cultural complexity and historical trauma. The single-text focus of this study, while necessarily limiting its generalizability, offers a valuable heuristic foundation for understanding genre adaptation in postcolonial settings. By concentrating intensively on one text, the analysis can uncover the intricate ways in which detective fiction conventions are reconfigured to engage with the specificities of colonial Bombay its plural legal traditions, intersecting religious and cultural identities, gendered power structures, and lingering historical traumas without the dilution that broader comparative sweeps sometimes entail. This close reading thus functions heuristically: it generates provisional concepts, interpretive frameworks, and critical questions (such as how hybrid subjectivities disrupt classic ratiocinative detection, or how spatially and legally plural contexts challenge Western genre expectations of singular justice) that can productively inform and guide subsequent scholarship. With clear applicability to comparative analyses in future work, this foundation invites extension to other postcolonial detective fictions whether in South Asia, Africa, the Caribbean, or elsewhere where authors similarly negotiate, subvert, or hybridize imported genre forms to articulate local experiences of modernity, law, gender, and decolonization.

### Implications

Sujata Massey’s novel, through its reworking of the detective fiction genre, provides a rich platform for interrogating systems of law, gender, and space in postcolonial contexts. Its narrative innovations and thematic concerns open new avenues for understanding how fiction can critique dominant ideologies and envision more inclusive models of justice.

First, at the theoretical level, the novel demonstrates the practical application of postcolonial and feminist theories to genre fiction. It affirms Homi Bhabha’s idea of hybridity as a narrative technique as well as a cultural state. Through spatial, legal, and identity-based hybridity, the novel illustrates the productive tensions and negotiations that occur in the “Third Space.” Similarly, it advances feminist legal theory by portraying law not as a detached apparatus but as a culturally contingent and gendered system. This reinforces the argument that justice, particularly for women in colonised societies, must be interpreted through an intersectional and context-sensitive lens.

From a literary perspective, this research suggests that detective fiction, often seen as formulaic or conservative, can serve as a potent site of subversion and transformation. Massey’s work challenges the rigid conventions of classic detective narratives by introducing ethical ambiguity, plural systems of knowledge, and a protagonist who navigates law and justice through empathy and negotiation. This has implications for how scholars and readers approach genre fiction, encouraging a reassessment of its capacity to engage with critical social and political issues.

In terms of social and cultural relevance, *A Murder on Malabar Hill* underscores the need for justice systems, both historical and contemporary, to be attuned to cultural diversity, gender dynamics, and social inequality. The novel’s critique of British colonial law and its limitations remains strikingly relevant today, especially in postcolonial societies still grappling with the legacies of legal pluralism, patriarchy, and systemic exclusion. Perveen Mistry’s hybrid approach to law and advocacy serves as a model for legal and ethical practice that is empathetic, situated, and responsive to marginalized voices.

Furthermore, the study offers significant pedagogical implications. Massey’s novel can be used effectively in university courses on postcolonial literature, gender and law, South Asian studies, and crime fiction. Its accessibility as a popular novel, paired with its deep engagement with serious theoretical and historical issues, makes it an ideal text for introducing students to interdisciplinary analysis. It also invites discussion about how literary texts can offer alternative forms of knowledge and critique, fostering critical thinking around issues of justice, power, and representation.

Lastly, this study opens up future research possibilities. Comparative analyses could be conducted between *A Murder on Malabar Hill* and other postcolonial detective novels that similarly rework genre conventions. Additionally, future work could explore how legal hybridity and spatial negotiation are represented in contemporary legal dramas or crime fiction in other postcolonial contexts such as Africa, the Caribbean, or Southeast Asia. The evolving role of female investigators in fiction across different cultural traditions is another promising area for further scholarly exploration.

In sum, the implications of this research affirm that *A Murder on Malabar Hill* is not only a compelling detective story but also a sophisticated commentary on the entangled legacies of colonialism, gender politics, and legal authority. It reveals that fiction, especially hybrid fiction, has the capacity not only to entertain but to provoke, critique, and imagine new social realities.

### Limitations

While this research offers a detailed and interdisciplinary analysis of Sujata Massey’s *A Murder on Malabar Hill*, it is important to acknowledge the limitations inherent in its scope, methodology, and focus. These limitations do not diminish the value of the findings but help define the boundaries within which they can be understood and applied.

First, this study is limited by its single-text focus. Although *A Murder on Malabar Hill* is a rich and representative example of postcolonial detective fiction, it is only one novel within a growing body of literature that engages with similar themes of legal hybridity, gendered justice, and spatial politics. A broader comparative study, including multiple texts either from Massey’s series or from other postcolonial contexts, could offer more generalizable insights into how the detective genre is being transformed globally. While this analysis centers on a single novel, *A Murder on Malabar Hill*, its scope is deliberately focused to allow in-depth exploration of hybrid mechanisms in one richly layered text. This case study holds significant heuristic value: it illuminates core processes of cultural hybridity, situated justice, and genre subversion in colonial-era detective fiction that can serve as a model or point of departure for future comparative research. For instance, these insights could be extended to other diasporic or postcolonial crime narratives (e.g., works by Abir Mukherjee or Anita Nair), enabling broader examination of how transnational authors reconfigure Western genre conventions across different colonial/postcolonial contexts.

This thesis, by design, emphasises depth over breadth and should therefore be seen as a focused case study rather than a comprehensive survey.

Second, the analysis is conducted within the framework of qualitative literary interpretation, which, while valuable for generating nuanced insights, is inherently subjective. Interpretive readings of character, space, law, and identity are shaped by the researcher’s theoretical orientations and critical positioning. Alternative readings using different critical lenses, such as Marxist theory, trauma theory, or reader-response criticism, could yield different conclusions about the novel’s meaning and significance.

Third, while this study engages with historical and legal contexts, it does so primarily through secondary sources and textual inference. It does not include archival research, interviews, or empirical fieldwork that might deepen the understanding of colonial legal practices, women’s lived experiences, or architectural representations in 1920s Bombay. A more historically grounded or interdisciplinary research design incorporating legal records, memoirs, or historical urban studies would offer a more robust empirical foundation for some of the claims made in this thesis.

In addition, the study focuses on literary representation rather than real-world legal systems, and therefore cannot claim to offer a definitive account of how hybrid legal frameworks functioned in practice during the colonial period. It analyses how the novel imagines and dramatises law, rather than how law was implemented or experienced historically. Thus, conclusions about justice, hybridity, or gender must be understood as interpretive readings of a fictional work, not empirical legal findings.

Lastly, the study centres on English-language literature written from a diasporic perspective. While Massey’s narrative is set in India, she writes from a transnational vantage point as an American author of South Asian descent. This position adds a valuable perspective, but it may also differ from texts written by Indian authors rooted in the subcontinent. Exploring regional or vernacular detective fiction might offer alternate portrayals of hybridity, justice, and gender.

Despite these drawbacks, by showing how *A Murder on Malabar Hill* both complicates and enhances the detective fiction tradition, this work makes a significant addition to the domains of postcolonial literature, feminist legal theory, and genre studies. The limitations listed here provide possible avenues for further investigation to broaden, contrast, and contextualize the knowledge produced by this targeted study.

### Future scope

This study, while focused on a single literary text, opens up several promising avenues for further research across literary studies, postcolonial theory, gender studies, and legal humanities. The themes and frameworks explored in *A Murder on Malabar Hill,* particularly legal hybridity, spatial negotiation, and feminist reinterpretations of detective fiction, provide a fertile foundation for broader scholarly inquiry.

One potential area for future research lies in conducting comparative studies of postcolonial detective fiction. While this thesis concentrates on Sujata Massey’s reworking of the genre in the context of colonial India, similar narrative transformations are occurring in postcolonial literatures from Africa, the Caribbean, and the Middle East. Examining how detective fiction evolves in different colonial and postcolonial settings could reveal both shared strategies and culturally specific innovations in how justice, authority, and identity are portrayed.

A related direction would involve a diachronic analysis of the Perveen Mistry series as a whole. This study analyses only *A Murder on Malabar Hill*, the first instalment, but Massey’s subsequent novels further develop Perveen’s character and continue to engage with changing legal, political, and gender dynamics in interwar India. Analyzing the full arc of the series would offer deeper insight into how hybrid identity and justice evolve within a historically grounded fictional world.

Another promising scope lies in cross-genre and intertextual research. Scholars might explore how elements of *A Murder on Malabar Hill* intersect with or depart from other genres such as historical fiction, legal thrillers, or feminist life writing. This would enrich our understanding of how genre blending operates as a form of narrative resistance and innovation in postcolonial literature.

Additionally, future research could incorporate a more interdisciplinary approach, combining literary analysis with legal history, feminist jurisprudence, urban studies, or anthropology. For example, archival research into real-life women lawyers, legal cases, and social reform movements in 1920s Bombay could help contextualize and historicise Massey’s fictional representation. Similarly, exploring actual court transcripts, colonial legal codes, or municipal planning records could deepen our understanding of how spatial and legal hybridity functioned materially during the period.

Another area of inquiry involves reader reception and popular discourse. As *A Murder on Malabar Hill* is also a commercially successful novel, researchers could investigate how different audiences, academic, general, South Asian, and diasporic, interpret Perveen Mistry’s hybrid identity and ethical framework. This could involve empirical methodologies such as reader surveys, book club studies, or content analysis of reviews and discussions.

Lastly, the novel offers a starting point for reimagining pedagogical practices in literary studies. Future work could examine how texts like *A Murder on Malabar Hill* can be used in classrooms to teach postcolonial theory, feminist legal critique, or spatial justice, especially to students unfamiliar with Indian history or legal pluralism.

## Conclusion

This article set out to examine how Sujata Massey’s *A Murder on Malabar Hill* reconfigures the conventions of detective fiction through the lenses of postcolonial theory, feminist legal critique, spatial analysis, and genre studies. Through a qualitative, interpretive approach grounded in close reading and interdisciplinary theory, the study has demonstrated that Massey’s novel is more than a historical crime story; it is a complex literary intervention that interrogates systems of law, gender, and cultural identity within a colonial framework.

The research established that Massey transforms the detective genre by embedding it within a richly hybrid space: 1920s colonial Bombay. As established, Bombay functions as epistemological terrain, spaces such as the courtroom, the zenana, and the British club are not neutral; they are culturally coded and ideologically contested. Perveen’s liminal position enables a situated, culturally responsive form of justice that negotiates colonial and indigenous frameworks.

One of the article’s key findings is the representation of legal hybridity, where British colonial law operates alongside Islamic and other indigenous personal laws. The novel dramatises the tensions between these systems, particularly as they impact women’s rights and legal access. Massey does not portray these legal systems as binary opposites but highlights the nuanced negotiations required to deliver justice in such a pluralistic environment. Perveen’s hybrid legal reasoning, grounded in empathy and cultural fluency, offers a model of feminist jurisprudence attuned to the lived realities of marginalized individuals.

Additionally, the study has shown that *A Murder on Malabar Hill* subverts traditional genre expectations. By privileging relational inquiry, multiple viewpoints, and ethical nuance over the genre’s conventional certainty and closure, Massey critiques both colonial-patriarchal structures and the epistemological limits of Western detection.

Bombay Massey’s narrative thus not only reclaims the detective form for postcolonial critique but also advances sociological insight into the co-constitution of race, gender, colonial legacies, and legal institutions, revealing fiction’s role in theorizing situated agency and institutional reform. Also, Urban sociologists interested in colonial and postcolonial cities might use the novel’s depiction of Bombay as a heuristic for examining how spatial hybridity structures social relations, mobility, and knowledge production in modern Indian metropolises. Broader postcolonial sociology could incorporate such fictional representations to theorize hybrid modernities, cultural translation, and resistance in transitional societies bridging literary close reading with sociological methods like ethnography, discourse analysis, or historical-comparative approaches. These interdisciplinary extensions would enrich sociological understandings of how popular cultural forms negotiate identity, authority, and social order, while affirming the value of fiction as evidence in studying enduring colonial legacies.

This thesis also emphasises that fiction, particularly hybrid genre fiction, is capable of producing critical knowledge about law, space, identity, and justice. By positioning a female protagonist within a colonial legal framework, Massey reclaims the detective narrative as a site of resistance, translation, and transformation. Perveen Mistry’s method of investigation is not only intellectual but deeply political and cultural, challenging dominant narratives while forging alternative paths to truth and equity. To conclude, *A Murder on Malabar Hill* is a wonderful example of how feminist and postcolonial writing can reimagine traditional genres to capture the complexities of hybrid cultures. It underscores the value of interdisciplinary literary analysis in uncovering the layered relationships between narrative, power, and culture. This study contributes to the growing body of scholarship that recognizes genre fiction as a legitimate and important site for theorizing justice, identity, and the politics of representation in postcolonial contexts.

## Data Availability

The original contributions presented in the study are included in the article/supplementary material, further inquiries can be directed to the corresponding author.

## References

[ref1] Ashcroft B. Griffiths G. Tiffinhfc H. (1989). The empire writes Back: Theory and practice in post-colonial literatures. London: Routledge.

[ref2] Bhabha H. K. (1994). The location of culture. London: Routledge.

[ref3] Chatterjee P. (1993). The nation and its fragments: Colonial and postcolonial histories. Princeton: Princeton University Press doi: 10.1515/9780691222232.

[ref4] Chettur S. K. (1940). Bombay Murder. Bombay: n.p.

[ref5] Foucault M. (1975). Discipline and punish: The birth of the prison. New York: Vintage Books.

[ref6] Ghosh S. (2014). The desi detective: popular fiction and Indian modernity. Econ. Polit. Wkly. 49, 59–64.

[ref7] Grewal I. (1996). Home and harem: Nation, gender, empire and the cultures of travel. Durham, NC: Duke University Press.

[ref8] Haraway D. (1988). Situated knowledges: the science question in feminism and the privilege of partial perspective. Feminist Stud. 14, 575–599. doi: 10.2307/3178066

[ref10] Kapur R. (2005). Erotic justice: Law and the new politics of postcolonialism. London: GlassHouse Press.

[ref11] Kidambi P. (2007). The making of an Indian Metropolis: Colonial governance and public culture in Bombay, 1890–1920. Aldershot: Ashgate.

[ref12] Kolsky E. (2010). Colonial justice in British India: White violence and the rule of law. Cambridge: Cambridge University Press.

[ref13] Lefebvre H. (1991). The production of space. Oxford: Blackwell.

[ref14] Massey S. (2018). A Murder on Malabar Hill. New York: Soho Crime.

[ref15] Mohanty C. T. (2003). Feminism without Borders: Decolonizing theory, practicing solidarity. Durham, NC: Duke University Press.

[ref16] Mukherjee M. (2003). Crime and empire in British India. New Delhi: Permanent Black.

[ref17] Mukherjee A. (2016). A Rising Man. London: Vintage Books.

[ref19] Mukherjee M. (2019). Postcolonial crime fiction. London: Routledge.

[ref20] Nair S. (1998). Caliban’s daughter: The tempest and the teapot. Minneapolis: University of Minnesota Press.

[ref22] Priestman M. (2003). Crime fiction: From Poe to the present. Devon: Northcote House.

[ref9] Rzepka C. J. Horsley L. (Ed.) (2010). A companion to crime fiction. Oxford: Wiley-Blackwell.

[ref24] Sathianandhan K. (1944). Detective Janaki. Madras: P. Varadachary and Co.

[ref25] Scaggs J. (2005). Crime Fiction. London: Routledge.

[ref27] Soja E. W. (1996). Thirdspace: Journeys to Los Angeles and other real-and-imagined places. Oxford: Blackwell.

[ref28] Ten Koraar N. (2005). “Postcolonial literature and the Western canon” in The Cambridge history of postcolonial literature. ed. Quayson A. (Cambridge: Cambridge University Press), 676–693.

[ref29] Todorov T. (1977). The typology of detective fiction. Howard R. The poetics of prose. Ithaca: Cornell University Press, 42–52.

[ref30] Young R. J. C. (1995). Colonial desire: Hybridity in theory, culture and race. London: Routledge.

